# Psychosocial predictors of COVID-19 infection in UK biobank (*N* = 104 201)

**DOI:** 10.1093/pubmed/fdad009

**Published:** 2023-04-12

**Authors:** Victor M Wauye, Frederick K Ho, Donald M Lyall

**Affiliations:** School of Health & Wellbeing, University of Glasgow, Scotland, UK; Department of Internal Medicine, Moi Teaching and Referral Hospital, Eldoret, Kenya; School of Health & Wellbeing, University of Glasgow, Scotland, UK; School of Health & Wellbeing, University of Glasgow, Scotland, UK

**Keywords:** COVID-19, predictors, psychosocial, UK Biobank

## Abstract

**Background:**

Since the outbreak of COVID-19, data on its psychosocial predictors are limited. We therefore aimed to explore psychosocial predictors of COVID-19 infection at the UK Biobank (UKB).

**Methods:**

This was a prospective cohort study conducted among UKB participants.

**Results:**

The sample size was *N* = 104 201, out of which 14 852 (14.3%) had a positive COVID-19 test. The whole sample analysis showed significant interactions between sex and several predictor variables. Among females, absence of college/university degree [odds ratio (OR) 1.55, 95% confidence interval (CI) 1.45–1.66] and socioeconomic deprivation (OR 1.16 95% CI 1.11–1.21) were associated with higher odds of COVID-19 infection, while history of psychiatric consultation (OR 0.85 95% CI 0.77–0.94) with lower odds. Among males, absence of college/university degree (OR 1.56, 95% CI 1.45–1.68) and socioeconomic deprivation (OR 1.12, 95% CI 1.07–1.16) were associated with higher odds, while loneliness (OR 0.87, 95% CI 0.78–0.97), irritability (OR 0.91, 95% CI 0.83–0.99) and history of psychiatric consultation (OR 0.85, 95% CI 0.75–0.97) were associated with lower odds.

**Conclusion:**

Sociodemographic factors predicted the odds of COVID-19 infection equally among male and female participants, while psychological factors had differential impacts.

## Introduction

Over 80% of COVID-19 cases are mild but can shed viral copies causing transmission of the disease,^1^ posing a public health challenge. However, most studies focus on clinical factors associated with severe COVID-19 outcomes.^2^^,^^3^ Studies published on psychosocial factors and COVID-19 reported on sociodemographic factors but are largely limited to severe COVID-19 outcomes.^4^^,^^5^ Defined as multidimensional constructs that influence well-being of a person, psychosocial factors such as anxiety, neuroticism, irritability, socioeconomic deprivation, level of education and ethnicity have been shown to influence health^6^^,^^7^ and play a significant role in the development of both physical and mental illness.^8^^,^^9^ Higher socioeconomic deprivation is reported to be associated with higher morbidity from both infectious and non-infectious diseases,^10^ and negative psychological factors such as depression and anxiety are associated with higher risk of developing medical conditions such as diabetes mellitus (DM^11^) and respiratory illnesses.^12^

This study therefore aimed to explore the role of psychosocial factors in predicting COVID-19 infection in the UK Biobank (UKB), hypothesizing that sociodemographic factors: non-white British ethnicity, college/university degree, socioeconomic deprivation, and psychological factors: depression, anxiety, neuroticism, loneliness, miserableness, irritability and history of psychiatric consultation predicted the odds of COVID-19 infection among the UKB participants, particularly around the first wave of the infections in Spring 2020.

## Methodology

### Study design and population

This study utilized secondary data provided by the UKB, a prospective cohort study that recruited volunteer participants between 2006 and 2010 and aimed to investigate determinants of disease among those aged 40–69 years until death.^13^*N* = 9 million adults whose contact information were gathered from the NHS were mailed, out of whom ⁓500 000 people were recruited. Data were generally collected using detailed touchscreen questionnaires.

### Primary outcome variable

The primary outcome was COVID-19 test result. COVID-19 data were provided by the Public Health Scotland, Public Health England and Secure Anonymized Information Linkage for the Scottish, English and Welsh data, respectively, and linked to the UKB database.^14^ The data contained the type of the specimen collected and the date of collection, the laboratory that processed the samples, the origin of the samples (collected from inpatient or not) and COVID-19 test result as positive or negative. Test samples were collected from nose or throat swabs and sometimes from the lower respiratory system in intensive care unit settings and analysed for COVID-19 reverse transcriptase-polymerase chain reaction (RT-PCR). The data covered the period from 16 March 2020 when the results started being released to the UKB to 31 December 2020. Among the participants who had undergone multiple COVID-19 tests and with different test results, any positive test outcome was treated as a positive case. Negative cases were considered negative if the test result was negative for the participants who had a single COVID-19 test, or negative for all the tests among those who underwent multiple tests. This study was carried out under UKB project number 17689.

### Predictor variables

Psychosocial predictor variables were taken during the UKB baseline assessments in 22 centers in England, Wales and Scotland from 2006 to 2010.^13^^,^^15^

#### Sociodemographic factors

Age and sex were self-reported; sex categorized as male and female. Ethnicity and level of education were self-reported and subsequently derived as White British and Non-white British, and with and without college/university degree, respectively. Level of deprivation was measured using Townsend Deprivation Scores, a measure of the level of district material deprivation in the UK, comprising four variables: percentage of people who own cars, stay in overcrowded areas, are unemployed and do not own homes.^16^ Individuals were assigned scores corresponding to their area post codes and area deprivation index. Higher scores indicated more socioeconomic deprivation.

#### Psychological factors

Seven psychological factors were explored. Neuroticism was measured using the revised version of the Eysenck Personality Questionnaire. Scores were summed into a continuous scale, with the higher values indicating more severe neuroticism. Depression, anxiety, loneliness, miserableness, irritability and history of psychiatric consultation were self-reported as ‘Yes’ or ‘No’. There is substantial evidence that these phenotypes, which are to some extent inter-correlated, have validity with regards subsequent health.^17–19^

#### Lifestyle factors

History of smoking was categorized as ever/never smoked, while history of alcohol intake as regular/non-regular drinker. Standing height was measured in centimeters using Seca 202 device, while weight was measured in kilograms (kg). Body mass index (BMI) was then constructed by dividing weight by squared height in meters (kg/m^2^) and classified into: underweight (<18.5 kg/m^2^), normal weight (18.5–24.9 kg/m^2^), overweight (25–29.9 kg/m^2^) and obese (≥30 kg/m^2^) according to World Health Organization (WHO) classification.^20^

#### Other comorbidities and biomarkers

Comorbidities such as DM, cancer, heart disease and hypertension were self-reported and/or confirmed by the medications that the participants were taking during assessments, where such medications were available. Blood pressure was taken with Omron 705 IT electronic BP monitor, for which two readings were taken and an average measurement calculated. Where automated measurement was not possible, a manual sphygmomanometer was used. Hypertension was then defined by BP more than or equal to 140/90 mmHg.^21^ Lung function was expressed as forced expiratory volume (FEV1). FEV1 is a measure of obstructive lung diseases such as chronic bronchitis/emphysema and asthma.^22^ It was measured through spirometry using a Vitalograph Pneumotrac 6800 equipment. Furthermore, handling of blood samples and laboratory parameters such as C-reactive protein (CRP), cholesterol levels and adult glycosylated hemoglobin (HbA1c) were extensively described in a different manual.^23^

### Statistical analysis

Statistical analysis was conducted using R statistical software version R-4.1.1. Continuous variables were described using mean/standard deviation (SD) for normally distributed data, and median/interquartile range (IQR) for skewed data. Categorical variables were described as frequencies/percentages.

Different models of logistic regression were used to explore the odds of COVID-19 infection. Model 1 involved univariate logistic regression. Model 2 was adjusted for baseline age and sex. Thereafter, multivariable analysis proceeded in two arms. For sociodemographic variables, model 3 was adjusted for psychological variables, while for psychological variables, model 3 was adjusted for sociodemographic variables. Models 4 and 5 involved adjustments for lifestyle factors and all covariates, respectively. Level of significance was set *P* < 0.05 with 95% confidence intervals. Sensitivity analysis was conducted using both complete case analysis and Hosmer-Lemeshow (HL) test of goodness of fit. Multicollinearity was tested using variance inflation factor (VIF).

## Results

### Results of the descriptive characteristics

Out of 502 490 UKB participants, 473 089 were alive on 16 March 2020. *N* = 104 201 had COVID-19 test results for the study period, out of whom 14 852 (14.25%) tested positive. The mean age of those who tested positive and negative was 52.86 (SD 8.41) and 57.53 (SD 7.96), respectively (Table 1).

**Table 1:** Whole-sample baseline characteristics

**Variables**		**COVID test result**	
**COVID-19 test results**		**Positive, *N* (%)**	**Negative, *N* (%)**
*N* (%)		14 852 (14.3)	89 349 (85.7)
**Demographic**			
Age (Years), Mean (SD)		52.86 (8.41)	57.53 (7.96)
Sex	Female, *N* (%)	7930 (14.2)	48 022 (85.8)
	Male, *N* (%)	6922 (14.3)	41 327 (85.7)
**Social variables**			
Non-white British, *N* (%)	No	12 470 (13.8)	78 220 (86.2)
	Yes	2330 (17.7)	10 797 (82.3)
College/university Degree, N (%)	Yes	3738 (11.9)	27 715 (88.1)
	No	10 926 (15.3)	60 667 (84.7)
Townsend score, Median (IQR)		−1.57 (7.68)	−2.13 (6.85)
**Psychological variables**			
Depression, N (%)	No	13 942 (14.3)	83 645 (85.7)
	Yes	910 (13.8)	5704 (86.2)
Anxiety, N (%)	No	14 650 (14.3)	88 077 (85.7)
	Yes	202 (13.7)	1272 (86.3)
Neuroticism Score, Median (IQR)		4 (5)	4 (5)
Loneliness	No	11 595 (14.1)	70 510 (85.9)
	Yes	2917 (14.6)	17 092 (85.4)
Miserableness	No	7618 (13.4)	49 189 (86.6)
	Yes	6891 (15.2)	38 355 (84.8)
Irritability	No	9760 (13.9)	60 685 (86.1)
	Yes	4350 (15.2)	24 339 (84.8)
Psychiatric Consultation	No	13 019 (14.4)	77 257 (85.6)
	Yes	1695 (12.9)	11 422 (87.1)
**Lifestyle variables**			
Smoking Status	No	8030 (14.8)	46 405 (85.2)
	Yes	6733 (13.7)	42 322 (86.3)
Regular Alcohol Drinking	No	4542 (15.9)	24 055 (84.1)
	Yes	9720 (13.6)	61 634 (86.4)
BMI	Normal	4085 (13.1)	27 040 (86.9)
	Underweight	56 (12.0)	411 (88.0)
	Overweight	6270 (14.3)	37 661 (85.7)
	Obese	4336 (15.5)	23 632 (84.5)
**Comorbidities**			
DM	No	13 936 (14.4)	83, 146 (85.6)
	Yes	824 (12.6)	5726 (87.4)
Bronchitis/Emphysema	No	14 739 (14.3)	88 628 (85.7)
	Yes	113 (13.5)	721 (86.5)
Cancer	No	13 909 (14.7)	80 995 (85.3)
	Yes	943 (10.1)	8354 (89.9)
Asthma	No	12 885 (14.2)	77 947 (85.8)
	Yes	1967 (14.7)	11 402 (85.3)
Heart Disease	No	14 164 (14.4)	83 986 (85.6)
	Yes	638 (11.1)	5107 (88.9)
HTN	No	11 147 (15.2)	62 185 (84.8)
	Yes	3655 (12.0)	26 908 (88.0)
**Biomarkers**			
CRP, Median (IQR)^a^		1.42 (2.26)	1.40 (2.22)
HbA1c, Mean (SD)^b^		35.93 (6.88)	36.51 (7.23)
Cholesterol, Mean (SD)^c^		5.58 (1.01)	(1.15)

^a^In mg/L.

^b^In mmol/mol.

^c^In mg/L.

In terms of infection, *N* = 12 470 (13.8%) of the White British versus *N* = 2330 (17.7%) of the Non-white British, and *N* = 10 926 (15.3%) of those without versus *N* = 3738 (11.9%) of those with college/university degree developed COVID-19 infection. The median Townsend score of those who tested positive was −1.57 (IQR 4.22), while of those who tested negative was −2.13 (IQR 4.22). Regarding psychological variables, *N* = 910 (13.8%) of those with depression, *N* = 202 (13.7%) of those with anxiety, *N* = 2917 (14.6%) of those with loneliness, *N* = 6891 (15.2%) of those with miserableness, *N* = 4350 (15.2%) of those with irritability and *N* = 1695 (12.9%) of those with history of psychiatric consultation developed COVID-19 infection (Table 1).

### Logistic regression results

#### Overall regression results

The whole sample analysis revealed that univariately, all sociodemographic variables examined were significantly associated with increased odds of infection. Among the psychological variables, higher baseline neuroticism, miserableness and irritability were associated with increased odds, while history of psychiatric consultation with reduced odds (Supplementary Table 1). On multivariable regression, absence of education degree and Townsend score remained significantly associated with increased odds: odds ratio (ORs) of 1.56 (1.48–1.64, < 0.0001) and 1.14 (1.10–1.17, < 0.0001), respectively, while loneliness and history of psychiatric consultation with reduced odds, ORs of 0.90 (0.84–0.96, 0.0019) and 0.85 (0.78–0.92, <0.0001), respectively (Supplementary Table 2).

The overall multivariable analysis, however, showed evidence of poor fit with *P*-values of 0.067, 0.057, 0.0063 and 0.0008 for HL groups 8, 10, 12 and 20, respectively. There was also significant interaction between sex and several predictor variables; sex with: education degree (*P*-value of 0.0138), ethnicity (0.0006), depression (0.0110), neuroticism (0.0060), miserableness (0.0051) and irritability (<0.0001), where COVID-19 was the outcome. Therefore, sub-analysis according to sex was conducted and the regression results presented as female-only and male-only results.

#### Female-only regression results

Regarding sociodemographic variables, being without degree and higher Townsend score were independently associated with the increased odds of COVID-19 infection, OR 1.55 [95% confidence interval (CI) 1.45–1.66, <0.0001] and OR 1.16 (95% CI 1.11–1.21, <0.0001), respectively. Regarding the psychological factors, only history of psychiatric consultation was independently associated with the reduced odds, OR 0.85 (95% CI 0.77–0.94, 0.0014) (Table 2). Descriptive characteristics and univariate analysis for the females were as in Supplementary Tables 3 and 4.

**Table 2:** Multivariable logistic regression outcomes of the females

	Model 2^a^		Model 3S^b^		Model 4^c^		Model 5^d^	
Predictors	OR	*P*-value	OR	*P*-value	OR	*P*-value	OR	*P*-value
Social								
Non-white British, Yes	1.03 (0.96, 1.11)	0.420	1.01 (0.93, 1.10)	0.752	0.99 (0.91, 1.08)	0.843	0.96 (0.87, 1.05)	0.371
No Degree, Yes	1.53 (1.44, 1.62)	<0.0001	1.55 (1.45, 1.65)	<0.0001	1.54 (1.44, 1.65)	<0.0001	1.55 (1.44, 1.66)	<0.0001
Townsend score (per SD)	1.15 (1.11, 1.18)	<0.0001	1.16 (1.12, 1.20)	<0.0001	1.15 (1.11, 1.19)	<0.0001	1.16 (1.11, 1.21)	<0.0001
**Psychological**			**Model 3P** ^**e**^					
Depression, Yes	0.98 (0.88, 1.10)	0.781	0.98 (0.88, 1.10)	0.775	0.98 (0.87, 1.10)	0.720	1.00 (0.89, 1.14)	0.881
Anxiety, Yes	0.97 (0.78, 1.20)	0.788	0.97 (0.78, 1.20)	0.791	0.99 (0.80, 1.24)	0.960	0.91 (0.71, 1.16)	0.430
Neuroticism (per SD)	1.04 (0.99, 1.09)	0.114	1.02 (0.97, 1.07)	0.391	1.01 (0.97, 1.06)	0.560	1.02 (0.97, 1.07)	0.529
Loneliness, Yes	0.96 (0.89, 1.04)	0.340	0.92 (0.85, 1.00)	0.042	0.93 (0.86, 1.01)	0.093	0.94 (0.87, 1.00)	0.051
Miserableness, Yes	1.07 (0.99, 1.14)	0.075	1.05 (0.98, 1.13)	0.180	1.05 (0.97, 1.13)	0.222	1.04 (0.96, 1.12)	0.355
Irritability, Yes	1.01 (0.94, 1.08)	0.868	1.03 (0.95, 1.10)	0.489	1.03 (0.96, 1.11)	0.441	1.04 (0.96, 1.13)	0.367
Psychiatric Cons., Yes	0.86 (0.79, 0.94)	0.0012	0.86 (0.79, 0.94)	0.0010	0.85 (0.77, 0.93)	0.0006	0.85 (0.77, 0.94)	0.0014

Adjusted for:

F—Social. P—Psychological. Cons. Consultation.

^a^Model 2: Age.

^b^Model 3S: Model 2 + Depression, Anxiety, Neuroticism, Loneliness, Miserableness, Irritability and Psychiatric Consultation.

^c^Model 4: Model 3 + Smoking Status, Regular Alcohol Intake and BMI Categories.

^d^Model 5: All covariates: Age, Depression, Anxiety, Neuroticism, Loneliness, Miserableness, Irritability, Psychiatric Consultation, Non-white British Ethnicity, College/university Degree, Townsend score, Smoking Status, Regular Alcohol Intake, BMI Categories, DM, Cancer, Bronchitis/Emphysema, Heart Disease, Asthma, HTN, CRP, HbA1c and Cholesterol.

^e^Model 3P: Model 2 + Non-white British Ethnicity, College/University Degree and Townsend score.

#### Male-only regression results

Among the males, absence of college/university degree and Townsend score were independently associated with the increased odds of COVID-19 infection, ORs of 1.56 (95% CI 1.45–1.68, <0.0001) and 1.12 (95% CI 1.07–1.16, <0.0001), respectively (Table 3). Regarding psychological factors, loneliness, OR 0.87 (95% CI 0.78–0.97, 0.011); irritability, OR 0.91 (95% CI 0.83–0.99, 0.029) and history of psychiatric consultation, OR 0.85 (95% CI 0.75–0.97, 0.0073) were independently associated with the reduced odds (Table 3). Descriptive characteristics and univariate analysis for the males were as in the Supplementary Tables 5 and 6.

**Table 3:** Multivariable logistic regression outcomes of the males

	Model 2^a^		Model 3S^b^		Model 4^c^		Model 5^d^	
Predictors	OR	*P*-value	OR	*P*-value	OR	*P*-value	OR	*P*-value
Social								
Non-white British, Yes	1.20 (1.11, 1.30)	<0.0001	1.12 (1.02, 1.23)	0.0166	1.09 (0.99, 1.20)	0.088	1.09 (0.99, 1.21)	0.090
No Degree, Yes	1.59 (1.49, 1.69)	<0.0001	1.65 (1.54, 1.76)	<0.0001	1.60 (1.50, 1.72)	<0.0001	1.56 (1.45, 1.68)	<0.0001
Townsend score (per SD)	1.11 (1.07, 1.15)	<0.0001	1.12 (1.08, 1.16)	<0.0001	1.11 (1.07, 1.16)	<0.0001	1.12 (1.07, 1.16)	<0.0001
**Psychological**			**Model 3P** ^**e**^					
Depression, Yes	0.92 (0.78, 1.08)	0.308	0.92 (0.78, 1.09)	0.345	0.92 (0.77, 1.09)	0.319	0.93 (0.77, 1.12)	0.431
Anxiety, Yes	0.85 (0.61, 1.17)	0.311	0.87 (0.63, 1.20)	0.396	0.86 (0.61, 1.20)	0.363	0.88 (0.62, 1.25)	0.472
Neuroticism (per SD)	1.02 (0.97, 1.07)	0.551	1.00 (0.95, 1.05)	0.936	1.02 (0.97, 1.08)	0.372	1.03 (0.98, 1.09)	0.274
Loneliness, Yes	0.92 (0.83, 1.01)	0.071	0.89 (0.80, 0.98)	0.0143	0.87 (0.78, 0.96)	0.0049	0.87 (0.78, 0.97)	0.011
Miserableness, Yes	1.02 (0.94, 1.11)	0.590	1.00 (0.92, 1.08)	0.930	1.00 (0.92, 1.08)	0.910	0.99 (0.90, 1.08)	0.798
Irritability, Yes	0.93 (0.86, 1.00)	0.052	0.94 (0.87, 1.02)	0.114	0.92 (0.85, 1.00)	0.0482	0.91 (0.83, 0.99)	0.0290
Psychiatric Cons., Yes	0.85 (0.76, 0.95)	0.0033	0.85 (0.76, 0.95)	0.0037	0.85 (0.76, 0.95)	0.005	0.85 (0.75, 0.96)	0.0073

Adjusted for:

S—Social. P—Psychological. Cons: Consultation.

^a^Model 2: Age.

^b^Model 3S: Model 2 + Depression, Anxiety, Neuroticism, Loneliness, Miserableness, Irritability and Psychiatric Consultation.

^c^Model 4: Model 3 + Smoking Status, Regular Alcohol Intake and BMI Categories.

^d^Model 5: All covariates: Age, Depression, Anxiety, Neuroticism, Loneliness, Miserableness, Irritability, Psychiatric Consultation, Non-white British Ethnicity, College/university Degree, Townsend score, Smoking Status, Regular Alcohol Intake, BMI Categories, Diabetes mellitus, Cancer, Bronchitis/Emphysema, Heart Disease, Asthma, Hypertension, CRP, HbA1c and Cholesterol.

^e^Model 3P: Model 2 + Non-white British Ethnicity, College/University Degree and Townsend score.

### Sensitivity analysis

Complete case analysis did not yield any statistically different results (Supplementary Tables 7 and 8). HL test of goodness of fit showed that the regression models used were of good fit with statistically nonsignificant *P*-values (Supplementary Table 9), and there was no multicollinearity, with all the VIFs being ˂5 (Supplementary Table 10).

## Discussion

### The main findings of this study

This study showed that absence of college/university degree and socioeconomic deprivation were independently associated with the higher odds of COVID-19 test positivity, and this did not substantively vary by sex. Psychological variables on the other hand were associated with the lower odds of COVID-19 test positivity, differentially according to sex. Notably, history of psychiatric consultation was associated with the lower odds equally among both female and the male participants, whereas loneliness and irritability were additionally associated with lower odds among male participants only.

### What is already known on this topic

Sociodemographic factors such as socioeconomic deprivation and lower levels of education have been shown to increase the risk of COVID-19 infection in previous literature.^2^^,^^4^^,^^5^^,^^24^ Rozenfeld *et al.* reported that socioeconomic deprivation increased the odds of COVID-19 infection by 10%.^24^ Chadeau-Hyam *et al.* reported that low education increased the odds of COVID-19 infection by 23%, OR 1.23 (95% CI 1.11–1.36),^25^ while Rozenfeld *et al.* reported an increased odds of 2%, OR 1.02 (95% CI 1.01–1.14).^24^

Psychological factors have been shown to predict poor health with regards to high risk behaviors and non-communicable diseases,^8^^,^^9^^,^^11^^,^^26^ although their role in infectious diseases such as COVID-19 is scarce, with the available literature being equivocal.^27–30^ Batty *et al.*^29^ and Lee *et al.*^27^ did not find any significant association between history of psychiatric consultation and COVID-19 infection. Taquet *et al.*^28^ and Wang *et al.*^30^ reported an increased risk, while Rozenfeld *et al.*^24^ reported that a diagnosis of a serious persistent mental condition reduced the odds of COVID-19 infection by 23%, OR 0.77 (95% CI 0.65–0.92).

### What this study adds

#### Sociodemographic factors

The finding that socioeconomic deprivation increased the odds of COVID-19 infection by ⁓14% was congruent with other published studies.^5^^,^^24^ The lack of the influence of sex, psychological factors, lifestyle factors and comorbidities on the effect of socioeconomic deprivation could be explained by the fact that the adverse effects of socioeconomic deprivation experienced in childhood may accumulate over one’s lifetime and predispose to ill health.^31–33^

Absence of college/university degree was associated with the increased odds of COVID-19 infection by ˃50%. Notably, the findings in this study revealed much higher odds than those reported by those reported in the previous literature. This could be attributed to the difference in the sample sizes. Furthermore, available evidence also shows that lower education and literacy is associated with low health literacy and health status.^34^^,^^35^ This may result in failure to understand health related information^36^ and negatively influence health seeking behavior.^35^ Notably, a lot of information regarding safety measures against COVID-19 such as social distancing and wearing of face masks were released and evolved during the outbreak. This could lead to difficulty in keeping up with such information especially among people with low level of education, hence increasing the risk of the infection. A COVID-19 Snapshot Monitoring Study in Germany showed that people with low level of education performed substantially lower than those with higher level of education with regards to adherence to social distance.^37^

#### Psychological factors

History of psychiatric consultation, which was used as a proxy to history of having mental health conditions was independently associated with the reduced odds of COVID-19 infection equally among the male and female participants. The available evidence for this relationship is scarce and mixed.^27–29^ It could be possible that people with the history of psychiatric consultation did not freely associate with others, hence protecting them from contracting COVID-19. With the current mixed evidence, further studies are still needed in this area.

This, to the best of our knowledge, was the first study to explore the effect of loneliness in predicting COVID-19 infection. Defined as a subjective perceived lack of social contact and not necessarily objective lack of social relations and contact,^38–40^ loneliness has been shown to predict health.^8^^,^^41–44^ There is some evidence that people with loneliness, from a loneliness-perpetuation perspective, are more likely to perceive the world as unsafe, act less prosocially and withdraw from others.^38^^,^^45–47^ Therefore, noting that COVID-19 is mainly spread by close contact with people who are infected,^48^^,^^49^ it could be possible that people with loneliness withdrew from others, hence avoiding social contacts and resulting in the protective effect observed in this study.

Finally, this was also the first study to explore irritability and COVID-19 infection, showing a protective association. Previous literature suggests that social support can improve psychological resilience^50^ and reduce the negative impacts of anger and irritability through positive anger disposition and coping mechanisms,^51^ hence positively influencing health. Although social support was not explored in this study, it could be possible that social support modified the effect of irritability in this study, hence the protective findings, though further studies are still needed.

### Sex differences

The observed sex differences could be explained by prior evidence that women and men have potential differential exposure and vulnerabilities to health conditions.^52–54^ Although effect sizes of most psychological variables examined were higher among women than men in this study, more psychological factors were significantly protective among men than women. This was an interesting finding noting that the risk of COVID-19 infection and severe outcome had been reported to be significantly higher among men than women in other studies.^3^^,^^5^^,^^25^

### Strengths and limitations

This study had the following strengths. First, to the best of our knowledge, it was the first study to examine the role of psychosocial factors in predicting the odds of COVID-19 infection in the general population, hence more applicable in providing evidence on how psychosocial factors influenced COVID-19 infection during the first year of its outbreak. Second, the large sample size used made it more generalizable. Third, utilizing the data from the UKB prospective cohort study minimized the likelihood of reverse causality. Fourth, COVID-19 test results were obtained from linkage of the UKB data with NHS. In addition, the samples were tested for the COVID-19 RT-PCR. This meant that the outcome variable in this study was accurately confirmed, hence reliable. Although false positive cases could still be possible, it was not a major concern since RT-PCR is the recommended diagnostic test for COVID-19 by the WHO.^55^

This study, however, had the following limitations. First, participants were mainly volunteers who would be healthier than the general population, hence would lead to selection bias. Selection bias could also be induced by including only participants with a test result in this study. Second, participants represented only 5.5% of the invited population, with significant under-representation of the non-white British ethnicity in particular, which affects generalizability. It is possible there are differences in risk factor/COVID-19 associations in non-White British sample, which this study was not statistically powered to test for. Third, baseline characteristics of the participants at the UKB were taken ˃10 years ago. Previous longitudinal data in UKB over mean 5 years suggests reasonable stability in individual differences such as cognitive health.^56^ Noting that baseline characteristics such as age, socioeconomic status and education change over time, it was possible that some of the participants were not correctly classified in their respective categories, hence misclassification bias. Fourth, COVID-19 vaccination status of the participants was not controlled for in this study. Noting that UK was among the first countries to roll out COVID-19 vaccination, failure to control for it would lead to a bias. Finally, using COVID-19 test results would mean that those who had undergone the test but were still awaiting the results and those who died of COVID-19 between January 2020 when the first case of COVID-19 was reported in the UK and 16 March 2020 when COVID-19 test results started being released to the UKB were excluded from analysis, which would underestimate the size effects.

## Conclusion

Sociodemographic factors significantly predicted COVID-19 infection at the UKB. These findings are important in the development of the primary prevention strategies. However, being new findings especially with regards to the effects of the psychological factors, more replication studies are needed before translation of these findings into policies.

## Conflict of Interest

None.

## Funding

Not applicable.

## Author Contributions

Conceived and designed the experiments: Victor M. Wauye & Donald M. Lyall.

Performed the experiments: Victor M. Wauye.

Facilitated acquisition of data: Donald M. Lyall; Frederick K. Ho.

Analysed the data: Victor M. Wauye.

Wrote the paper: Victor M. Wauye & Donald M. Lyall.

Critically revised the manuscript for important intellectual content: All the authors.

## Ethics Approval

UKB received ethical approval from the North-West Multicenter Research Ethics Committee, Community of Health Index Advisory Group in Scotland and Patient Information Advisory Group in England and Wales. Participants provided full informed consent to participate and for publication of the research findings. This study was also covered by the generic ethical approval for UKB studies from the NHS National Research Ethics Service (approval letter dated 17 June 2011, Ref 11/NW/0382).^57^

## Data availability statement

UK Biobank is an open-access resource available to verified researchers upon application (http://www.ukbiobank.ac.uk/).

## Supplementary Material

Wauye_Suppl_fdad009

## Notes

**Victor M. Wauye**, MPH, Medical Doctor (MBChB)

**Frederick K. Ho**, Lecturer in Public Health

**Donald M. Lyall**, Lecturer in Public Health
