# Supplementary material for: Psychosocial predictors of COVID-19 infection in UK biobank (N = 104 201)
Source: J Public Health (Oxf). 2023 Apr 12;45(3):560–8. doi: 10.1093/pubmed/fdad009 (PMC10470346; doi:10.1093/pubmed/fdad009)
Supplement: Wauye_Suppl_fdad009 [file wauye_suppl_fdad009.docx]

**Supplementary Information: Psychosocial Predictors of Covid-19 Infection at the UK Biobank**

Table 1: Whole Sample Univariate Analysis Results

|  | | OR | 95% CI | P-value |
| --- | --- | --- | --- | --- |
| Demographic Factors | | | | |
| Age (Per Year) | | 0.93 | 0.93, 0.94 | <0.0001 |
| Male Sex | | 1.01 | 0.98, 1.05 | 0.424 |
| Social Factors | | | | |
| Non-White British, Yes | | 1.35 | 1.29, 1.42 | <0.0001 |
| No Degree , Yes | | 1.34 | 1.28, 1.39 | <0.0001 |
| Tds (Per Sd) | | 1.16 | 1.15, 1.18 | <0.0001 |
| Psychological Factors | | | | |
| Depression, Yes | | 0.96 | 0.89, 1.03 | 0.235 |
| Anxiety, Yes | | 0.95 | 0.82, 1.11 | 0.544 |
| Neuroticism Score (Per Sd) | | 1.06 | 1.04, 1.08 | <0.0001 |
| Loneliness, Yes | | 1.04 | 0.99, 1.08 | 0.097 |
| Miserableness, Yes | | 1.16 | 1.12, 1.20 | <0.0001 |
| Irritability, Yes | | 1.11 | 1.07, 1.16 | <0.0001 |
| Psychiatric Consultation, Yes | | 0.88 | 0.83, 0.93 | <0.0001 |
| Lifestyle Factors | | | | |
| Smoking Status, Yes | | 0.92 | 0.89, 0.95 | <0.0001 |
| Regular Alcohol Intake, Yes | | 0.84 | 0.80, 0.87 | <0.0001 |
| Bmi Categories | Normal (Ref) |  |  |  |
|  | Underweight | 0.90 | 0.68, 1.19 | <0.0001 |
|  | Overweight | 1.10 | 1.06, 1.15 |  |
|  | Obese | 1.21 | 1.16, 1.27 |  |
| Comorbidities and Biomarkers | | | | |
| Dm, Yes | | 0.85 | 0.80, 0.93 | <0.0001 |
| Bronchitis/Emphysema, Yes | | 0.94 | 0.77, 1.15 | 0.559 |
| Cancer, Yes | | 0.66 | 0.61, 0.70 | <0.0001 |
| Asthma, Yes | | 1.04 | 0.99, 1.10 | 0.103 |
| Heart Disease, Yes | | 0.74 | 0.68, 0.81 | <0.0001 |
| HTN, Yes | | 0.76 | 0.73, 0.79 | <0.0001 |
| CRP (Per SD) | | 1.00 | 0.99, 1.02 | 0.622 |
| HbA1c (Per SD) | | 0.91 | 0.89, 0.93 | <0.0001 |
| Cholesterol (Per SD) | | 0.94 | 0.92, 0.96 | <0.0001 |

Table 2: Multivariable Regression Results of the Whole Sample

|  | Model 2^a^ | | Model 3S^b^ | | Model 4^d^ | | Model 5^e^ | |
| --- | --- | --- | --- | --- | --- | --- | --- | --- |
| Predictors | **OR** | **P-value** | **OR** | **P-value** | **OR** | **P-value** | **OR** | **P-value** |
| Social |  |  |  |  |  |  |  |  |
| Non-White British, Yes | 1.10 (1.05, 1.16) | 0.0002 | 1.06 (1.00, 1.13) | 0.061 | 1.03 (0.97, 1.10) | 0.308 | 1.02 (0.95, 1.09) | 0.614 |
| No Degree, Yes | 1.55 (1.49, 1.62) | <0.0001 | 1.60 (1.52, 1.67) | <0.0001 | 1.57 (1.50, 1.65) | <0.0001 | 1.56 (1.48, 1.64) | <0.0001 |
| TDS (per SD) | 1.13 (1.10, 1.16) | <0.0001 | 1.14 (1.11, 1.17) | <0.0001 | 1.13 (1.10, 1.16) | <0.0001 | 1.14 (1.10, 1.17) | <0.0001 |
|  |  |  |  |  |  |  |  |  |
| Psychological |  |  | **Model 3P^c^** |  |  |  |  |  |
| Depression, Yes | 0.97 (0.88, 1.06) | 0.495 | 0.97 (0.89, 1.06) | 0.531 | 0.96 (0.87, 1.06) | 0.418 | 0.99 (0.89, 1.09) | 0.771 |
| Anxiety, Yes | 0.93 (0.78, 1.11) | 0.448 | 0.94 (0.79, 1.13) | 0.508 | 0.95 (0.79, 1.14) | 0.566 | 0.89 (0.73, 1.09) | 0.277 |
| Neuroticism (per SD) | 1.03 (0.99, 1.06) | 0.129 | 1.01 (0.98, 1.05) | 0.567 | 1.02 (0.98, 1.05) | 0.339 | 1.02 (0.98, 1.06) | 0.249 |
| Loneliness, Yes | 0.95 (0.89, 1.01) | 0.076 | 0.91 (0.86, 0.97) | 0.0025 | 0.91 (0.85, 0.97) | 0.0021 | 0.90 (0.84, 0.96) | 0.0019 |
| Miserableness, Yes | 1.04 (0.99, 1.10) | 0.109 | 1.02 (0.97, 1.08) | 0.376 | 1.02 (0.97, 1.08) | 0.456 | 1.01 (0.96, 1.07) | 0.655 |
| Irritability, Yes | 0.97 (0.92, 1.02) | 0.196 | 0.98 (0.93, 1.04) | 0.548 | 0.98 (0.93, 1.03) | 0.441 | 0.97 (0.92, 1.03) | 0.387 |
| Psychiatric Cons., Yes | 0.86 (0.80, 0.92) | <0.0001 | 0.85 (0.80, 0.91) | <0.0001 | 0.85 (0.79, 0.91) | <0.0001 | 0.85 (0.78, 0.92) | <0.0001 |

Adjusted for:

^a^Model 2: Age.

^b^Model 3S: Model 2 + Depression, Anxiety, Neuroticism, Loneliness, Miserableness, Irritability and Psychiatric Consultation.

^c^Model 3P: Model 2 + Non-White British Ethnicity, College/university Degree and TDS.

^d^Model 4: Model 3 + Smoking Status, Regular Alcohol Intake and BMI Categories.

^e^Model 5: All covariates: Age, Depression, Anxiety, Neuroticism, Loneliness, Miserableness, Irritability, Psychiatric Consultation, Non-White British Ethnicity, College/university Degree, TDS, Smoking Status, Regular Alcohol Intake, BMI Categories, DM, Cancer, Bronchitis/Emphysema, Heart Disease, Asthma, HTN, CRP, HbA1c and Cholesterol.

Cons: Consultation.

Table 3: Descriptive Analysis Females

| **Variables^a^** | | | **Covid Test Result (n= 55,952)** | |
| --- | --- | --- | --- | --- |
| **Covid-19 Test Results** | | | **Positive, N (%)** | **Negative, N (%)** |
| N (%) | | | 7,930 (14.17) | 48,022 (85.83) |
| **Demographic** | | | | |
| Age (Years), Mean (SD) | | | 52.5 (8.25) | 57.2 (7.97) |
| **Social Variables** | | | | |
| Non-White British | | Yes | 1,215 (16.7) | 6,068 (83.3) |
|  | | No | 6,688 (13.8) | 41,811 (86.2) |
| College/University Degree | | No | 5,863 (15.0) | 33,287 (85.0) |
|  | | Yes | 1,971 (12.1) | 14,258 (87.9) |
| TDS, Median (IQR) | | | -1.55 (4.64) | -2.10 (4.19) |
| **Psychological Variables** | | | | |
| Depression | | No | 7,294 (14.1) | 44,255 (85.9) |
|  | | Yes | 636 (14.4) | 3,767 (85.6) |
| Anxiety | | No | 7,790 (14.2) | 47,197 (85.8) |
|  | | Yes | 140 (14.5) | 825 (85.5) |
| Neuroticism Score, Median (IQR) | | | 5 (5) | 4 (5) |
| Loneliness | | No | 5,920 (14.0) | 36,385 (86.0) |
|  | | Yes | 1,841 (14.7) | 10,673 (85.3) |
| Miserableness | | No | 3,476 (12.9) | 23,442 (87.1) |
|  | | Yes | 4,287 (15.3) | 23,672 (84.7) |
| Irritability | | No | 5,255 (13.5) | 33,681 (86.5) |
|  | | Yes | 2,279 (16.0) | 11,969 (84.0) |
| Psychiatric Consultation | | No | 6,839 (14.3) | 40,958 (85.7) |
|  | | Yes | 1,014 (13.1) | 6,704 (86.9) |
| **Lifestyle Variables** | | | | |
| Smoking Status | | No | 4,576 (14.3) | 27,375 (85.7) |
|  | | Yes | 3,303 (14.0) | 20,332 (86.0) |
| Regular Alcohol Drinking | | No | 2,975 (15.4) | 16,386 (84.6) |
|  | | Yes | 4,639 (13.6) | 29,596 (86.4) |
| BMI | Normal | | 2,759 (13.6) | 17,570 (86.4) |
|  | Underweight | | 46 (12.0) | 336 (88.0) |
|  | Overweight | | 2,885 (14.2) | 17,489 (85.8) |
|  | Obese | | 2,203 (15.2) | 12,318 (84.8) |
| **Comorbidities** | | | | |
| DM | | No | 7,651 (14.3) | 45,486 (85.7) |
|  | | Yes | 324 (12.3) | 2,315 (87.7) |
| Bronchitis/Emphysema | | No | 7,858 (14.2) | 47,625 (85.8) |
|  | | Yes | 72 (15.4) | 397 (84.6) |
| Cancer | | No | 7,309 (14.6) | 42,712 (85.4) |
|  | | Yes | 621 (10.6) | 5,310 (89.5) |
| Asthma | | No | 6,772 (14.1) | 41,319 (85.9) |
|  | | Yes | 1,158 (14.7) | 6,703 (85.3) |
| Heart Disease | | No | 7,703 (14.2) | 46,380 (85.8) |
|  | | Yes | 200 (11.7) | 1,509 (88.3) |
| HTN | | No | 6,175 (15.0) | 35,038 (85.0) |
|  | | Yes | 1,728 (11.9) | 12,851 (88.1) |
| **Biomarkers** | | |  |  |
| CRP, Median (IQR) | | | 1.49 (2.60) | 1.47 (2.47) |
| HbA1c, Mean (SD) | | | 35.37 (6.13) | 36.14 (6.48) |
| Cholesterol, Mean (SD) | | | 5.67 (1.09) | 5.85 (1.13) |

^a^Percentages of the categorical variables were computed per the total numbers that tested

positive and those that tested negative in each category.

Table 4: Univariable Regression Outcomes of the Females

|  | | **OR** | **95% CI** | **P - Value** |
| --- | --- | --- | --- | --- |
| **Demographic Factors** | | | | |
| Age (per year) | | 0.93 | 0.93 – 0.94 | <0.0001 |
| **Social Factors** | | | | |
| Non-White British, Yes | | 1.25 | 1.17 – 1.34 | <0.0001 |
| No Degree, Yes | | 1.27 | 1.21 – 1.35 | <0.0001 |
| TDS (per SD) | | 1.17 | 1.14 – 1.20 | <0.0001 |
| **Psychological Factors** | | | | |
| Depression, Yes | | 1.02 | 0.94 – 1.12 | 0.59 |
| Anxiety, Yes | | 1.03 | 0.86 – 1.23 | 0.763 |
| Neuroticism Score (per SD) | | 1.09 | 1.06 – 1.12 | <0.0001 |
| Loneliness, Yes | | 1.06 | 1.00 – 1.12 | 0.043 |
| Miserableness, Yes | | 1.22 | 1.16 – 1.28 | <0.0001 |
| Irritability, Yes | | 1.22 | 1.16 – 1.29 | <0.0001 |
| Psychiatric Consultation, Yes | | 0.91 | 0.84 – 0.97 | 0.0062 |
| **Lifestyle Factors** | | | | |
| Smoking Status, Yes | | 0.97 | 0.93 – 1.02 | 0.246 |
| Regular Alcohol Intake, Yes | | 0.86 | 0.82 – 0.91 | <0.0001 |
| BMI Categories | Normal (Ref) |  |  |  |
|  | Underweight | 0.87 | 0.64 – 1.19 | 0.387 |
|  | Overweight | 1.05 | 0.99 – 1.11 | 0.086 |
|  | Obese | 1.14 | 1.07 – 1.21 | <0.0001 |
| **Comorbidities and Biomarkers** | | | | |
| DM, Yes | | 0.84 | 0.75 – 0.95 | 0.0045 |
| Bronchitis/Emphysema, Yes | | 1.10 | 0.85 – 1.41 | 0.462 |
| Cancer, Yes | | 0.68 | 0.63 – 0.75 | <0.0001 |
| Asthma, Yes | | 1.05 | 0.99 – 1.13 | 0.126 |
| Heart Disease, Yes | | 0.80 | 0.69 – 0.93 | 0.0031 |
| HTN, Yes | | 0.76 | 0.72 – 0.81 | <0.0001 |
| CRP (per SD) | | 1.00 | 1.00 – 1.01 | 0.235 |
| HbA1c (per SD) | | 0.85 | 0.82 – 0.89 | <0.0001 |
| Cholesterol (Per SD) | | 0.85 | 0.82 – 0.87 | <0.0001 |

Table 5: Descriptive Analysis Males

| **Variables^a^** | | | **Covid Test Result (n= 48,249)** | |
| --- | --- | --- | --- | --- |
| **Covid-19 Test Results** | | | **Positive, N (%)** | **Negative, N (%)** |
| N (%) | | | 6,922 (14.35) | 41,327 (85.65) |
| **Demographic** | | | | |
| Age (Years), Mean (SD) | | | 53.25 (8.58) | 57.97 (7.93) |
| **Social Variables** | | | | |
| Non-White British | | Yes | 1,115 (19.1) | 4,729 (80.9) |
|  | | No | 5,782 (13.7) | 36,409 (86.3) |
| College/University Degree | | Yes | 1,767 (11.6) | 13,457 (88.4) |
|  | | No | 5,063 (15.6) | 27,380 (84.4) |
| TDS, Median (IQR) | | | -1.59 (4.8) | -2.15 (4.28) |
| **Psychological Variables** | | | | |
| Depression | | No | 6,648 (14.4) | 39,390 (85.6) |
|  | | Yes | 274 (12.4) | 1,937 (87.6) |
| Anxiety | | No | 6,860 (14.4) | 40,880 (85.6) |
|  | | Yes | 62 (12.2) | 447 (87.8) |
| Neuroticism Score, Median (IQR) | | | 3 (5) | 6 (5) |
| Loneliness | | No | 5,675 (14.3) | 34,125 (85.7) |
|  | | Yes | 1,076 (14.4) | 6,419 (85.6) |
| Miserableness | | No | 4,142 (13.9) | 25,747 (86.1) |
|  | | Yes | 2,604 (15.1) | 14,683 (84.9) |
| Irritability | | No | 4,505 (14.3) | 27,004 (85.7) |
|  | | Yes | 2,071 (14.3) | 12,370 (85.7) |
| Psychiatric Consultation | | No | 6,180 (14.5%) | 36,299 (85.5) |
|  | | Yes | 681 (12.6%) | 4,718 (87.4) |
| **Lifestyle Variables** | | | | |
| Smoking Status | | No | 3,454 (15.4%) | 19,030 (84.6) |
|  | | Yes | 3,430 (13.5%) | 21,990 (86.5) |
| Regular Alcohol Drinking | | No | 1,567 (17.0%) | 7,669 (83.0) |
|  | | Yes | 5,081 (13.7%) | 32,038 (86.3) |
| BMI | Normal | | 1,326 (12.3%) | 9,470 (87.7) |
|  | Underweight | | 10 (11.8%) | 75 (88.2) |
|  | Overweight | | 3,385 (14.4%) | 20,172 (85.6) |
|  | Obese | | 2,133 (15.9%) | 11,314 (84.1) |
| **Comorbidities** | | | | |
| DM | | No | 6,375 (14.5) | 37,660 (85.5) |
|  | | Yes | 500 (12.8) | 3,411 (87.2) |
| Bronchitis/Emphysema | | No | 6,881 (14.4) | 41,003 (85.6) |
|  | | Yes | 41 (11.2) | 324 (88.8) |
| Cancer | | No | 6,600 (14.7) | 38,283 (85.3) |
|  | | Yes | 322 (9.6) | 3,044 (9.6) |
| Asthma | | No | 6,113 (88.6) | 36,628 (85.7) |
|  | | Yes | 809 (14.7) | 4,699 (85.3) |
| Heart Disease | | No | 6,461 (14.7) | 37,606 (85.3) |
|  | | Yes | 438 (10.9) | 3,598 (89.1) |
| HTN | | No | 4,972 (15.5) | 27,147 (84.5) |
|  | | Yes | 1,927 (12.1) | 14,057 (87.9) |
| **Biomarkers** | | |  |  |
| CRP, Median (IQR) | | | 1.36 (1.91) | 1.33 (1.95) |
| HbA1c, Mean (SD) | | | 36.57 (7.60) | 36.94 (7.98) |
| Cholesterol, Mean (SD) | | | 5.48 (1.22) | 5.42 (1.14) |

^a^Percentages of the categorical variables were computed per the total numbers that tested

positive and those that tested negative in each category.

Table 6: Univariate Regression Outcomes of the Males

|  | | **OR** | **95% CI** | **P - Value** |
| --- | --- | --- | --- | --- |
| **Demographic Factors** | | | | |
| Age (per year) | | 0.93 | 0.93 – 0.94 | <0.0001 |
| **Social Factors** | | | | |
| Non-White British, Yes | | 1.48 | 1.38 – 1.59 | <0.0001 |
| No Degree, Yes | | 1.41 | 1.34 – 1.49 | <0.0001 |
| TDS (per SD) | | 1.16 | 1.13 – 1.19 | <0.0001 |
| **Psychological Factors** | | | | |
| Depression, Yes | | 0.84 | 0.74 – 0.95 | 0.0074 |
| Anxiety, Yes | | 0.83 | 0.63 – 1.08 | 0.162 |
| Neuroticism Score (per SD) | | 1.03 | 1.00 – 1.06 | 0.0417 |
| Loneliness, Yes | | 1.01 | 0.94 – 1.08 | 0.825 |
| Miserableness, Yes | | 1.10 | 1.05 – 1.16 | 0.0003 |
| Irritability, Yes | | 1.00 | 0.95 – 1.06 | 0.901 |
| Psychiatric Consultation, Yes | | 0.85 | 0.78 – 0.92 | 0.0001 |
| **Lifestyle Factors** | | | | |
| Smoking Status, Yes | | 0.86 | 0.82 – 0.90 | <0.0001 |
| Regular Alcohol Intake, Yes | | 0.78 | 0.73 – 0.83 | <0.0001 |
| BMI Categories | Normal (Ref) |  |  |  |
|  | Underweight | 0.95 | 0.49 – 1.85 | 0.885 |
|  | Overweight | 1.20 | 1.12 – 1.28 | <0.0001 |
|  | Obese | 1.35 | 1.25 – 1.45 | <0.0001 |
| **Comorbidities and Biomarkers** | | | | |
| DM, Yes | | 0.87 | 0.79 – 0.95 | 0.0038 |
| Bronchitis/Emphysema, Yes | | 0.75 | 0.54 – 1.04 | 0.090 |
| Cancer, Yes | | 0.61 | 0.55 – 0.69 | <0.0001 |
| Asthma, Yes | | 1.03 | 0.95 – 1.12 | 0.443 |
| Heart Disease, Yes | | 0.71 | 0.64 – 0.79 | <0.0001 |
| HTN, Yes | | 0.75 | 0.71 – 0.79 | <0.0001 |
| CRP (per SD) | | 0.99 | 0.97 – 1.02 | 0.591 |
| HbA1c (per SD) | | 0.96 | 0.93 – 0.98 | 0.0005 |
| Cholesterol (per SD) | | 1.05 | 1.03 – 1.08 | <0.0001 |

Table 7: Complete Case Analysis Females

|  | Model 2^a^ | | Model 3S^b^ | | Model 4^d^ | | Model 5^e^ | |
| --- | --- | --- | --- | --- | --- | --- | --- | --- |
| Predictors | **OR** | **P-value** | **OR** | **P-value** | **OR** | **P-value** | **OR** | **P-value** |
| Social |  |  |  |  |  |  |  |  |
| Non-White British, Yes | 0.97 (0.88, 1.06) | 0.453 | 0.97 (0.88, 1.07) | 0.519 | 0.96 (0.87, 1.05) | 0.371 | 0.96 (0.87, 1.05) | 0.371 |
| No Degree, Yes | 1.58 (1.47, 1.69) | <0.0001 | 1.58 (1.47, 1.69) | <0.0001 | 1.55 (1.44, 1.66) | <0.0001 | 1.55 (1.44, 1.66) | <0.0001 |
| TDS (per SD) | 1.16 (1.11, 1.20) | <0.0001 | 1.16 (1.12, 1.21) | <0.0001 | 1.16 (1.11, 1.21) | <0.0001 | 1.16 (1.11, 1.21) | <0.0001 |
|  |  |  |  |  |  |  |  |  |
| Psychological |  |  | **Model 3P^c^** |  |  |  |  |  |
| Depression, Yes | 1.02 (0.90, 1.15) | 0.728 | 1.02 (0.90, 1.15) | 0.783 | 1.01 (0.89, 1.14) | 0.977 | 1.01 (0.89, 1.14) | 0.882 |
| Anxiety, Yes | 0.93 (0.73, 1.18) | 0.544 | 0.91 (0.71, 1.16) | 0.451 | 0.91 (0.71, 1.16) | 0.426 | 0.91 (0.71, 1.16) | 0.430 |
| Neuroticism (per SD) | 1.04 (0.99, 1.09) | 0.170 | 1.02 (0.96, 1.07) | 0.562 | 1.02 (0.97, 1.07) | 0.484 | 1.02 (0.97, 1.07) | 0.529 |
| Loneliness, Yes | 0.96 (0.88, 1.05) | 0.350 | 0.92 (0.85, 1.01) | 0.076 | 0.92 (0.84, 1.00) | 0.049 | 0.92 (0.84, 1.00) | 0.051 |
| Miserableness, Yes | 1.06 (0.98, 1.14) | 0.156 | 1.04 (0.96, 1.13) | 0.311 | 1.04 (0.96, 1.12) | 0.367 | 1.04 (0.96, 1.12) | 0.355 |
| Irritability, Yes | 1.01 (0.94, 1.10) | 0.724 | 1.04 (0.96, 1.12) | 0.379 | 1.04 (0.96, 1.10) | 0.389 | 1.04 (0.96, 1.13) | 0.367 |
| Psychiatric Cons., Yes | 0.85 (0.77, 0.94) | 0.0018 | 0.85 (0.77, 0.94) | 0.0014 | 0.85 (0.77, 0.94) | 0.0013 | 0.85 (0.77, 0.94) | 0.0014 |

Adjusted for:

^a^Model 2: Age.

^b^Model 3S: Model 2 + Depression, Anxiety, Neuroticism, Loneliness, Miserableness, Irritability and Psychiatric Consultation.

^c^Model 3P: Model 2 + Non-White British Ethnicity, College/university Degree and TDS.

^d^Model 4: Model 3 + Smoking Status, Regular Alcohol Intake and BMI Categories.

^e^Model 5: All covariates: Age, Depression, Anxiety, Neuroticism, Loneliness, Miserableness, Irritability, Psychiatric Consultation, Non-White British Ethnicity, College/university Degree, TDS, Smoking Status, Regular Alcohol Intake, BMI Categories, DM, Cancer, Bronchitis/Emphysema, Heart Disease, Asthma, HTN, CRP, HbA1c and Cholesterol.

S - Social. P - Psychological. Cons. Consultation.

Table 8: Complete Case Analysis Males

|  | Model 2^a^ | | Model 3S^b^ | | Model 4^d^ | | Model 5^e^ | |
| --- | --- | --- | --- | --- | --- | --- | --- | --- |
| Predictors | **OR** | **P-value** | **OR** | **P-value** | **OR** | **P-value** | **OR** | **P-value** |
| Social |  |  |  |  |  |  |  |  |
| Non-White British, Yes | 1.12 (1.01, 1.24) | <0.0349 | 1.11 (1.00, 1.23) | 0.0430 | 1.10 (0.99, 1.22) | 0.066 | 1.09 (0.99, 1.21) | <0.090 |
| No Degree, Yes | 1.61 (1.50, 1.73) | <0.0001 | 1.62 (1.50, 1.74) | <0.0001 | 1.56 (1.45, 1.68) | <0.0001 | 1.56 (1.45, 1.68) | <0.0001 |
| TDS (per SD) | 1.11 (1.07, 1.16) | <0.0001 | 1.12 (1.08, 1.17) | <0.0001 | 1.12 (1.07, 1.16) | <0.0001 | 1.12 (1.07, 1.16) | <0.0001 |
|  |  |  |  |  |  |  |  |  |
| Psychological |  |  | **Model 3P^c^** |  |  |  |  |  |
| Depression, Yes | 0.95 (0.79, 1.14) | 0.557 | 0.94 (0.79, 1.13) | 0.530 | 0.93 (0.77, 1.11) | 0.411 | 0.93 (0.77, 1.12) | 0.431 |
| Anxiety, Yes | 0.86 (0.61, 1.22) | 0.400 | 0.87 (0.61, 1.24) | 0.450 | 0.87 (0.61, 1.24) | 0.441 | 0.88 (0.62, 1.25) | 0.472 |
| Neuroticism (per SD) | 1.03 (0.97, 1.09) | 0.328 | 1.02 (0.96, 1.08) | 0.509 | 1.03 (0.97, 1.09) | 0.313 | 1.03 (0.98, 1.09) | 0.274 |
| Loneliness, Yes | 0.92 (0.83, 1.02) | 0.122 | 0.89 (0.80, 1.09) | 0.029 | 0.87 (0.79, 0.97) | 0.0134 | 0.87 (0.78, 0.97) | 0.0112 |
| Miserableness, Yes | 1.03 (0.94, 1.12) | 0.505 | 1.00 (0.92, 1.09) | 0.994 | 0.99 (0.91, 1.08) | 0.811 | 0.99 (0.90, 1.08) | 0.798 |
| Irritability, Yes | 0.91 (0.83, 0.99) | 0.0273 | 0.92 (0.85, 1.01) | 0.066 | 0.91 (0.83, 0.99) | 0.0284 | 0.91 (0.83, 0.99) | 0.029 |
| Psychiatric Cons., Yes | 0.86 (0.76, 0.97) | 0.0115 | 0.85 (0.75, 0.96) | 0.0087 | 0.85 (0.75, 0.95) | 0.0015 | 0.85 (0.75, 0.96) | 0.0073 |

Adjusted for:

^a^Model 2: Age.

^b^Model 3S: Model 2 + Depression, Anxiety, Neuroticism, Loneliness, Miserableness, Irritability and Psychiatric Consultation.

^c^Model 3P: Model 2 + Non-White British Ethnicity, College/university Degree and TDS.

^d^Model 4: Model 3 + Smoking Status, Regular Alcohol Intake and BMI Categories.

^e^Model 5: All covariates: Age, Depression, Anxiety, Neuroticism, Loneliness, Miserableness, Irritability, Psychiatric Consultation, Non-White British Ethnicity, College/university Degree, TDS, Smoking Status, Regular Alcohol Intake, BMI Categories, DM, Cancer, Bronchitis/Emphysema, Heart Disease, Asthma, HTN, CRP, HbA1c and Cholesterol.

S – Social. P - Psychological. Cons. Consultation.

.

Table 9: HL Test Results of the Final Sex-specific Regression Models

| HL Group | **P-values** | |
| --- | --- | --- |
|  | **Females** | **Males** |
| 8 | 0.078 | 0.852 |
| 10 | 0.052 | 0.342 |
| 12 | 0.061 | 0.416 |
| 20 | 0.179 | 0.745 |

Table 10: Variance Inflation Factors of the Covariates

|  | **Females** | | **Males** | |
| --- | --- | --- | --- | --- |
| **Variable** | **GVIF** | **GVIF^(1/(2*Df))^** | **GVIF** | **GVIF^(1/(2*Df))^** |
| NonCaucasian | 1.08 | 1.04 | 1.08 | 1.04 |
| No Degree | 1.07 | 1.04 | 1.06 | 1.03 |
| Townsend | 1.10 | 1.05 | 1.09 | 1.05 |
| Age | 1.33 | 1.15 | 1.21 | 1.00 |
| Depression | 1.17 | 1.08 | 1.17 | 1.08 |
| Anxiety | 1.03 | 1.01 | 1.03 | 1.01 |
| Neuroticism | 2.79 | 1.67 | 2.93 | 1.71 |
| Loneliness | 1.41 | 1.19 | 1.39 | 1.18 |
| Miserable | 1.67 | 1.29 | 1.77 | 1.33 |
| Irritable | 1.47 | 1.21 | 1.53 | 1.24 |
| Seen a Psychiatrist | 1.18 | 1.08 | 1.20 | 1.10 |
| Smoking Status | 1.05 | 1.02 | 1.06 | 1.03 |
| Regular Drinking | 1.10 | 1.05 | 1.07 | 1.03 |
| BMI Cat | 1.25 | 1.04 | 1.14 | 1.02 |
| DM | 1.53 | 1.24 | 1.73 | 1.32 |
| Cancer | 1.01 | 1.01 | 1.02 | 1.01 |
| Heart Disease | 1.06 | 1.03 | 1.12 | 1.06 |
| HTN | 1.16 | 1.08 | 1.17 | 1.08 |
| Asthma | 1.02 | 1.01 | 1.01 | 1.01 |
| Bronchitis | 1.01 | 1.00 | 1.00 | 1.00 |
| HbA1c | 1.65 | 1.29 | 1.71 | 1.31 |
| Cholesterol | 1.14 | 1.07 | 1.17 | 1.08 |
| CRP | 1.12 | 1.06 | 1.04 | 1.02 |
